# Pullout Behaviour and Influencing Mechanisms of Desert Plant Roots in Clayey Sand During Thawing

**DOI:** 10.3390/plants14182876

**Published:** 2025-09-16

**Authors:** Xiaofei Yang, Qinglin Li, Shuailong Yu, Pengrui Feng, Meixue Zhang, Wenjuan Chen, Guang Yang

**Affiliations:** 1 College of Water Resources and Construction Engineering, Shihezi University, Shihezi 832000, China; 13731701887@163.com (X.Y.); 18298145517@163.com (S.Y.); drfxj666@163.com (P.F.); m13031338205@163.com (M.Z.); mikeyork@163.com (G.Y.); 2 Key Laboratory of Cold and Arid Regions Eco-Hydraulic Engineering of Xinjiang Production & Construction Corps, Shihezi 832000, China; 3 College of Science, Shihezi University, Shihezi 832000, China; chenwj513@163.com

**Keywords:** thawing process, root–soil interface, *Alhagi sparsifolia*, peak pullout force, ecological engineering

## Abstract

In cold and arid regions, the mechanical properties and influencing mechanisms of the root–soil interface during the thawing stage remain poorly understood. This study focuses on *Alhagi sparsifolia* root–clayey sand composites to investigate the effects of temperature (−10 °C to 25 °C), initial soil water content (4–12%), and naturally varying root diameter (4.50–5.05 mm) on root pullout behaviour, and integrates endoscopic macro-observation, environmental scanning electron microscopy (ESEM), soil water migration tests, and nuclear magnetic resonance (NMR) techniques to reveal the dominant influencing mechanisms. Key findings reveal the following: (1) An increase in soil water content from 4% to 12%, and a temperature rise from −10 °C to 25 °C led to a maximum reduction in the average peak pullout force (F_T_) of roots exceeding 95%. (2) There is a non-monotonic relationship between root diameter and pull-out force, which can be attributed to two distinct failure modes: a newly observed failure mode known as root bark peeling, occurring under high soil moisture conditions (≥8%), and a commonly observed failure mode referred to as partial soil detachment, occurring under low soil moisture conditions (≤6%). (3) The coupling effects of temperature and water content reveal that the increase in temperature predominantly contributes to strength loss (>63%) during the ice–water phase transition (−10 °C to 0 °C), while soil water content primarily influences root pullout behaviour in the liquid water stage (5 °C to 25 °C). (4) As the temperature rises, in soils with low water content (4–6%), the reinforcing effect of roots appears to stabilize at −1 °C, whereas in soils with high water content (8–12%), stabilization occurs only beyond 5 °C. These findings enhance the understanding of root–soil interactions in thawing environments and provide a theoretical basis for soil bioengineering techniques aimed at slope stabilization in cold and arid regions.

## 1. Introduction

Slope failure and soil erosion are major eco-geological disasters faced globally [1,2]. In cold and arid regions such as northwest China, frequent freeze–thaw cycles reduce the mechanical properties of slope soils [3,4]. During the freezing period, ice formation induces frost-heave [5,6]; while during thawing, meltwater softens the soil, triggering hazards such as landslides, collapses, and erosion [7,8]. These processes severely threaten slope stability and infrastructure safety, underscoring the urgent need for effective ecological restoration strategies [9].

The soil bioengineering technique is an economical, environmentally friendly, and effective measure for sustainable ecological restoration [10,11]. In addition to the hydrological effects of the above-ground parts of plants—such as canopy interception of rainfall, litter suppression of runoff, and reduction in soil water evaporation [12,13,14]—plant roots serve as anchors that significantly enhance overall slope stability through shallow reinforcement and deep anchoring effects [15,16]. When slope soils undergo shear or sliding, particularly in the case of shallow landslides (i.e., landslides with a basal failure plane located in the range of 1–2 m soil depth), plant roots can penetrate potential slip surfaces and are subjected to complex loading conditions [17,18]. As root systems deform with the soil and reach a critical angle, their reinforcing effect gradually shifts from bending resistance to pullout resistance. In this stage, the frictional force between roots and soil is usually weaker than the tensile strength of the root; as a result, it causes root slippage rather than rupture [19]. At this stage, the bonding characteristics of the root–soil interface become the key factor in determining slope stability. Therefore, root pullout resistance is widely regarded as a key parameter for evaluating root reinforcement and the mechanical response at the root–soil interface, and an important entry point for revealing the reinforcement mechanism of root–soil composite systems [20,21]. In recent years, extensive experimental and modelling studies have been conducted by many researchers to investigate root pullout responses with various plant types, root morphological characteristics, and varying soil conditions.

In related studies, in situ pullout tests are routinely used to investigate the mechanical behaviour of the root–soil interface under natural conditions. Fan et al. (2021) [22] found that increasing soil water content significantly reduced the pullout resistance of paper mulberry (*Broussonetia papyrifera*) roots. In contrast, root diameter and root length showed a positive correlation with pullout resistance. The mechanical performance of the root–soil interface was quantitatively assessed through soil–root bond strength. Yang et al. (2021) [23] conducted pullout tests on alfalfa (*Medicago sativa* L.) roots and showed that root surface area has a greater influence on peak pullout resistance than root diameter and length. Their comparison of pulling angles indicated that vertical pullout tests more effectively assessed root reinforcement under controlled conditions. Alam et al. (2021) [24] investigated the pullout characteristics of four plant species using an In Situ Smart Testing Apparatus (ISTA) and revealed the regulatory role of soil porosity, water content, and compaction in the Soil Root Bonding Strength (SRBS). To control experimental variables and explore root–soil interface mechanisms precisely and deeply, researchers have extensively employed pullout tests using reconstituted root–soil samples to study root reinforcement mechanisms. This approach follows the pioneering concept of root reinforcement proposed by Waldron and Dakessian (1981) [25], later extended by Pollen and Simon (2005) [26], who incorporated tensile strength and soil–root cohesion into stability models. Schwarz et al. (2011) [27] demonstrated that root tortuosity and branching significantly enhance both peak pullout force and displacement. Giadrossich et al. (2013) [28] further quantified the collective mechanical interactions among neighbouring roots, while Cohen et al. (2011) [29] employed a fibre bundle model to highlight failure sequences in mixed-diameter root networks. Meijer et al. (2019) [30] introduced analogue roots to examine the interaction of axial tension and bending. At finer scales, De Baets et al. (2020) [31] utilised CT scanning to investigate how root hairs increase cohesion at the root–soil interface. Mao et al. (2023) [32] examined alfalfa roots across growth stages, proposing a dynamic model to predict time-varying mechanical effects of vegetation. Most recently, Houette et al. (2024) [33] employed 3D-printed biomimetic roots to isolate morphological influences, confirming that total root surface area significantly enhances pullout resistance.

Existing research on root pullout has yielded substantial results, providing a theoretical foundation for root pullout testing and soil-reinforcement mechanism analysis, but these studies have all been conducted on root–soil composites under normal temperature conditions, without considering root pullout behaviour during thawing at subzero temperatures. In cold and arid regions, however, root systems and root–soil composites on slopes inevitably undergo a thawing process [34,35], and this process is bound to affect the soil-reinforcement capacity and underlying mechanisms of roots. Considering these aspects, the current study focuses on understanding the variation patterns and internal influencing mechanisms of root pullout behaviour during critical freeze–thaw stages, especially the thawing process, in desert vegetation. To this end, *Alhagi sparsifolia*, a desert plant with a well-developed root system in cold and arid regions [36,37] and clayey sand were selected as the research objects. A self-developed root pullout testing system tailored for thawing conditions was used to investigate the effects of temperature, root diameter, and soil water content on the pullout behaviour of *A. sparsifolia* roots during the thawing process; meanwhile, both endoscopic imaging and environmental scanning electron microscopy (ESEM) were used to analyse the failure characteristics at the root–soil interface. Furthermore, soil water migration tests and nuclear magnetic resonance (NMR) techniques were applied to characterize soil water distribution, ice–water phase states, and pore characteristics. These methods jointly reveal the root reinforcement mechanisms of *A. sparsifolia* in clayey sand during thawing, providing a theoretical basis for optimising slope protection techniques and ecological restoration practices using desert vegetation in cold and arid regions.

## 2. Materials and Methods

### 2.1. Test Material

**Figure 1:**
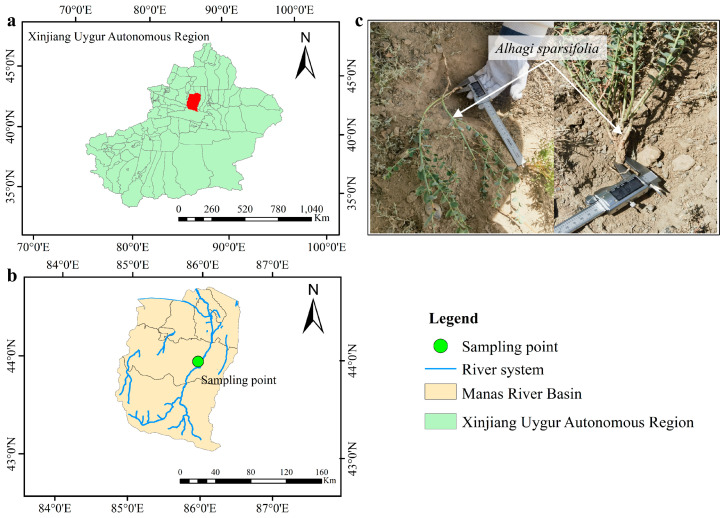
Geographic location of the study area and sampling point in the Manas River Basin, Xinjiang, China. (**a**) Location of the Manas River Basin within Xinjiang; (**b**) distribution of sampling point and river system in the basin; (**c**) field sampling and root selection of *Alhagi sparsifolia*.

The study site is located on slopes in proximity to the Manas River in the northern foothills of the Tianshan Mountains, Xinjiang, China, an area covered with native wild vegetation (Figure 1). This region has a typical mid-temperate continental arid to semi-arid climate, with cold winters and hot summers. The average annual precipitation is 237.7 mm, and the mean annual temperature is 8.7 °C. The maximum snow depth reaches 0.25 m, and the maximum frozen soil depth is approximately 0.75 m. Soil freeze–thaw processes primarily occur from November to March.

In this region, *Alhagi sparsifolia* is a dominant and ecologically important native shrub widely distributed across desert slopes. It has a dense and deep root system with strong drought resistance and salt tolerance, making it highly suitable for reinforcing shallow soils and restoring degraded slopes in cold and arid areas. These characteristics have been confirmed by previous studies [38,39]. Therefore, *A. sparsifolia* roots were selected for this study. Root samples of *A. sparsifolia* were collected from the previously described study site. Sampling was conducted on non-engineered slopes with a natural gradient of approximately 30°, free from anthropogenic disturbance such as roads or infrastructure. Roots were excavated manually at depths of 0–50 cm to preserve structural integrity. The basic physical properties of the root samples are summarised in Table 1. These values were obtained following the procedures described by Gao et al. (2023) [40]. Specifically, natural water content was calculated from the mass loss after air-drying the roots to constant weight. Tissue density was determined by dividing the air-dried mass by the volume of the dried root, measured using a root scanner. Maximum water absorption was calculated by comparing the fully saturated mass and the air-dried mass of the same root sample. Maximum longitudinal and radial shrinkage were determined as the percentage reductions in root length and diameter, respectively, from the saturated to the air-dried state.

**Figure 2:**
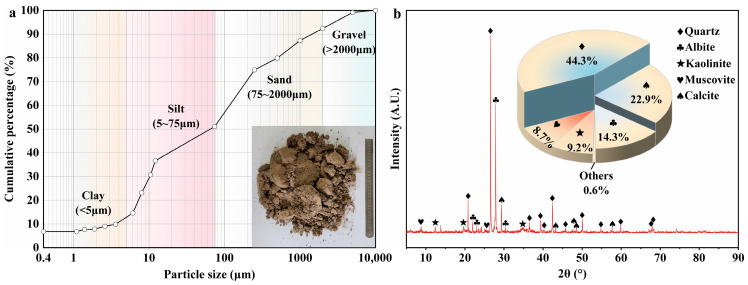
Basic test results of the test soil: (**a**) particle size distribution curve; (**b**) XRD pattern.

The soil used in the experiment was shallow soil from the native habitat of *A. sparsifolia* roots. According to the Chinese national standard (GB/T 50123−2019) [41], the natural water content and dry density of the soil were measured as 8.15% and 1.576 g/cm^3^, respectively. Impurities were removed from the collected soil samples, which were then air-dried and rolled; particles larger than 10 mm were excluded to facilitate laboratory testing. The particle size distribution was determined using a combined sieve–hydrometer analysis, as shown in Figure 2a. The results indicate that the test soil contains approximately 12.7% clay, 38.3% silt, 41.3% sand, and 7.7% gravel. Standard compaction tests indicated an optimum water content of 11.9% and a maximum dry density of 1.97 g/cm^3^. As shown in Figure 2b, X-ray diffraction (XRD) analysis of the test soil revealed that the mineral composition of the soil sample consists of quartz (44.3%), calcite (22.9%), albite (14.3%), kaolinite (9.2%), and muscovite (8.7%). The basic physical properties of the soil are summarised in Table 1 and identified as clayey sand (SC) by the Unified Soil Classification System (USCS).

### 2.2. Test Scheme

**Figure 3:**
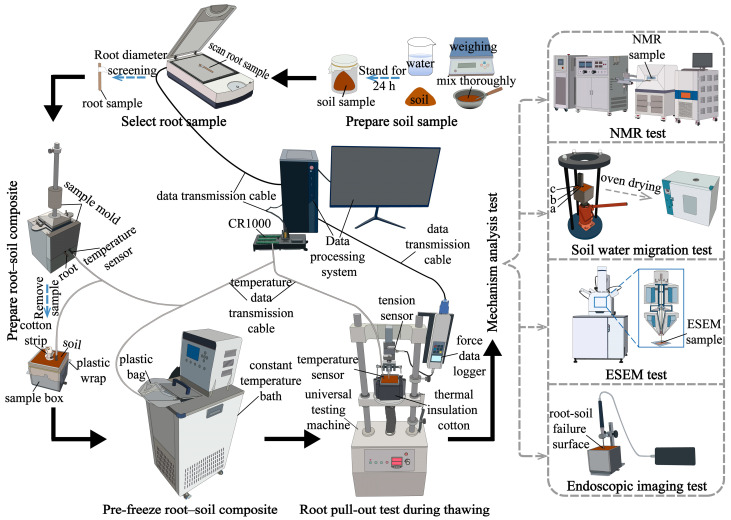
Workflow of the testing scheme.

As shown in Figure 3, the experimental procedure consisted of the following sequential steps: preparation of the soil sample, selection of root sample, preparation of root–soil composite, pre-freezing of the root–soil composite, root pullout test during thawing, and mechanism analysis tests. The mechanism analysis tests included four components: endoscopic imaging test, environmental scanning electron microscopy (ESEM) test, soil water migration test, and nuclear magnetic resonance (NMR) test. Previous studies have applied endoscopy to evaluate the root–soil contact state within biopores [42], used ESEM to observe the microstructure of soil in the presence of roots [43,44], and employed NMR to quantitatively analyse unfrozen water content and pore distribution characteristics in frozen soil [45,46,47].

**Figure 4:**
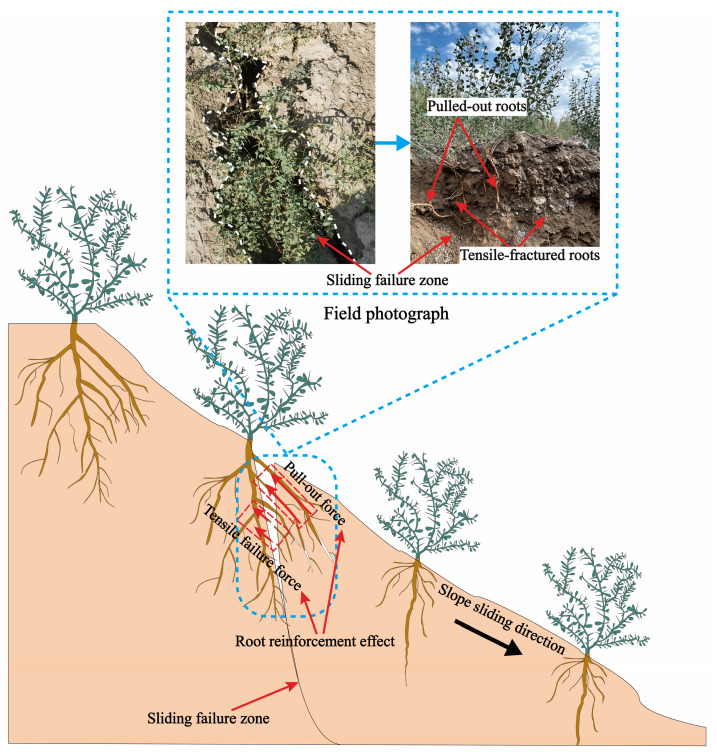
Two-dimensional schematic diagram of roots failure modes during slope failure.

Previous studies have shown that vertical pullout tests are best suited for evaluating the reinforcing capacity of roots [23]. The pullout resistance is only slightly affected by pulling speed, and slower speeds tend to produce greater fluctuations in measured resistance [48]. Therefore, vertical pullout tests were employed in this study to evaluate root pullout resistance, with a pulling speed set at 20 mm/min. Most current studies have demonstrated that root tensile strength increases with root diameter [49,50,51,52,53]. Moreover, there exists a root diameter threshold that dictates whether the root fails in tension or is pulled out [22,54]. Specifically, during slope failure, the coarse roots of *A. sparsifolia* located near the soil surface possess greater tensile strength than the root–soil frictional resistance, resulting in pullout failure. In contrast, the fine roots located in deeper soil layers tend to fail in tension as their tensile strength is lower compared to the root–soil frictional force [23]. As illustrated in the 2D schematic in Figure 4, the coarse roots of *A. sparsifolia* near the surface primarily experience pullout failure during slope failure, which was also confirmed by field photographs. To replicate this reinforcing mechanism under experimental conditions, relatively straight and coarse root segments were selected for testing.

In this study, root segments with diameters ranging from 4.50 mm to 5.05 mm were used. Considering the irreproducibility of root diameter [23], the root diameters were further subdivided into six diameter classes: 4.50–4.55 mm, 4.55–4.65 mm, 4.65–4.75 mm, 4.75–4.85 mm, 4.85–4.95 mm, and 4.95–5.05 mm. Based on the internal dimensions of the custom-built square sample box (80 mm × 80 mm × 80 mm), the root embedment depth was set to 80 mm. The clamping length was determined by the maximum gripping capacity of the tensile testing machine clamps, which was 30 mm. To minimise disruption of the root–soil composite during clamping, a 20 mm root section was reserved between the bottom of the clamp and the soil surface. Therefore, the total length of the root segments used in this test was 130 mm. Using the soil temperature data collected by sensors during thawing in the study area along with meteorological station records, six temperature gradients were established for the simulated root pullout tests during the thawing process. Based on the natural water content and dry density of the soil, five initial soil water content gradients were designed, with a uniform dry density of 1.576 g/cm^3^.

To ensure comprehensive evaluation of the key environmental and morphological factors influencing root–soil interaction during thawing, the combinations of root diameter, temperature, and soil water content were systematically designed, as summarised in Table 2. To further explore the mechanisms underlying root pullout behaviour during thawing, four complementary tests were conducted after the mechanical tests, including endoscopic imaging, environmental scanning electron microscopy (ESEM), soil water migration tests, and nuclear magnetic resonance (NMR) analyses. These tests targeted both macro- and micro-scale changes at the root–soil interface. The detailed design of the mechanism analysis tests is shown in Table 3, providing a framework to link observable failure patterns with internal water redistribution and microstructural evolution.

### 2.3. Sample Preparation

#### 2.3.1. Root–Soil Composites for Pullout Testing

The soil samples collected from the study area were crushed by rolling, oven-dried at 105 °C, and passed through a 10 mm sieve. To ensure the target soil dry density of 1.576 g/cm^3^ in each root–soil composite, the required mass of oven-dried soil was calculated based on the internal volume of the sample box (512 cm^3^). Accordingly, 807 g of dry soil (1.576 g/cm^3^ × 512 cm^3^) was weighed for each sample. After uniformly spraying the pre-calculated amount of water corresponding to the initial water content specified in the test design, the moistened soil was thoroughly mixed, sealed in airtight bags, and left to stand for 24 h to achieve uniform moisture distribution.

**Figure 5:**
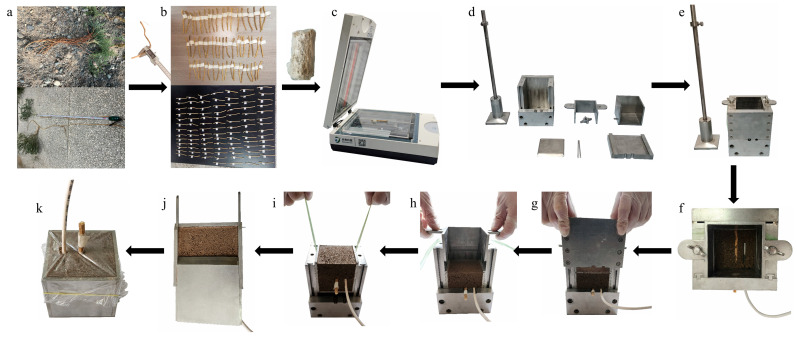
Preparation of *A. sparsifolia* root–soil composites: (**a**) initial field selection of root segments; (**b**) preparation of root samples; (**c**) final root selection; (**d**) components of the custom-built sample preparation device; (**e**) assembly of the preparation device; (**f**) insertion of root and temperature sensor; (**g**–**j**) removal of the root–soil composite and assembly of the sample box; (**k**) completed sample covered with plastic wrap for preservation.

To ensure consistency and minimize experimental error, relatively straight and undamaged *A. sparsifolia* roots (i.e., roots with undamaged bark and continuous cylindrical shape) were selected in the field (Figure 5a). The root diameter was preliminarily measured using a digital vernier caliper (accuracy 0.01 mm), and samples with diameters between 4.50 and 5.05 mm were trimmed to 130 mm in length. A label was affixed at 80 mm from the root end to define the analysis section (Figure 5b). To preserve root freshness, all selected samples were wrapped in plastic film and stored at 4 °C. Before root–soil composite preparation, roots were scanned using a root scanner (Phantom 9980XL, D&J (Shanghai) Technology Co., Ltd., Shanghai, China) to determine the average diameter of the 80 mm long section. Root samples that matched the designated diameter ranges were selected for testing (Figure 5c).

A custom-built device developed by the authors’ research group was used to prepare the root–soil composites (Figure 5d–k). The device consists of a detachable square sample box (internal dimensions: 80 mm × 80 mm × 80 mm), a forming shell, a compaction hammer, and several fixing components. The sample box includes one removable side panel and one fixing slot. During preparation, the fixing slot was first inserted into the forming shell and secured in place (Figure 5e). A two-layer compaction method was employed: the first layer of prepared soil was compacted to half the height of the fixing slot, the surface was roughened, and the root and a temperature sensor were inserted into the soil (Figure 5f). After completing the compaction of the second soil layer, the root–soil composite was removed and the sample box was assembled (Figure 5g–j). Finally, the composite sample was covered with plastic wrap to prevent drying (Figure 5k).

#### 2.3.2. ESEM Samples

ESEM testing samples were extracted from a root–soil composite at −10 °C after measuring the pullout resistance in the soil with 12% water content, but prior to pulling the root out. From this composite, three approximately cubic samples with typical dimensions close to 10 mm × 10 mm × 10 mm (length × width × height) were cut. These three rectangular samples included a composite sample with the pulled root (with root), a composite sample with the unpulled root (with root), and a soil sample containing both disturbed and undisturbed zones after root pullout (without root).

#### 2.3.3. NMR Soil Samples

NMR system sample tubes were cylindrical plastic rings made of Teflon, with an inner diameter and height of 25 mm and 60 mm, respectively. Based on the tube dimensions and the designed soil dry density of 1.576 g/cm^3^, oven-dried soil was weighed to prepare five soil samples with initial water contents of 4%, 6%, 8%, 10%, and 12%. The prepared soil samples were compacted into the sample tubes in a single step using plastic sleeves and compaction rods printed by a 3D printer (Pro3 HS, Shanghai Fusion Tech Co., Ltd., Shanghai, China). To ensure uniform water distribution within the cylindrical soil samples, they were wrapped with plastic film and left to stand in the dark for 24 h.

### 2.4. Test Method

#### 2.4.1. Pullout Test During the Thawing Process

In this study, root–soil composites were frozen using a constant-temperature bath with a temperature range from −30 °C to 100 °C and at an accuracy of 0.05 °C. Temperature data from the sensor embedded in the root–soil composite were recorded in real-time at 1 s intervals using a data logger (CR1000, Campbell Scientific, Logan, UT, USA). A digital force gauge (maximum load 1000 N, accuracy 0.1 N) and a tension testing machine were used to perform root pullout tests.

For the freezing process, the root–soil composite was placed in a plastic bag and submerged in the circulating liquid of the constant-temperature bath. The circulating liquid temperature of the bath was set according to six thawing temperature gradients established (Table 4). The root–soil composite was pre-frozen for 4 h. Once the temperature stabilised, the composite was removed, and the exposed root and the sample box were wrapped with cotton strips and thermal insulation cotton, respectively. The root–soil composite was then mounted on the tension testing machine, and the sample was allowed to thaw at room temperature until it reached the target temperature specified in the test design (Table 4), immediately followed by the root pullout test. The root pullout behaviour (peak pullout force and soil–root bond strength) under each test condition was obtained. The soil–root bond strength ($\tau_{r}$) was estimated using Equation (1) [22].

(1) $$\tau_{r}=F_{P}/\left(\pi DL\right)$$

In the equation, $F_{P}$ represents the peak pullout force of the root in soil (N), D is the average root diameter (mm), and L is the root embedment depth (mm). In this test, the root embedment depth was fixed at 80 mm, and the soil–root bond strength was only affected by the peak pullout force and root diameter.

#### 2.4.2. Endoscopic Imaging Test

Endoscopic imaging followed the pullout tests of roots with diameters ranging from 4.95 to 5.05 mm. The tested samples included 30 soil specimens in which roots were pulled out under six thawing temperatures (−10 °C, −5 °C, −1 °C, 0 °C, 5 °C, and 25 °C) and five initial soil water contents (4%, 6%, 8%, 10%, and 12%). An industrial endoscope (lens diameter: 3.2 mm; length: 60 mm; resolution: 1280 × 720) was inserted into the void created by root pullout to capture images of the root–soil failure interface.

#### 2.4.3. ESEM Test

ESEM was used for microscopic analysis of root–soil bonding and failure mechanisms. The cryo-stage of the Quattro SEM (Quattro, Thermo Fisher Scientific, Waltham, MA, USA) was set to −10 °C. Although the samples were not extracted directly in a frozen state, they were promptly placed into sealed boxes and refrozen at −10 °C for 12 h in a constant-temperature chamber to stabilise their structure. The samples were then surface gold-coated and observed at the cryo-stage, ensuring that they remained in a frozen state throughout the coating and imaging process.

#### 2.4.4. Soil Water Migration Test

For the soil water migration test, samples were selected after the root pullout tests. Three parallel samples were selected from each combination of designed thawing temperature (−10 °C, −5 °C, −1 °C, 0 °C, 5 °C, 25 °C) and initial soil water content (4%, 6%, 8%, 10%, 12%), resulting in a total of 90 soil samples for testing. A custom-made sampler was used for soil extraction, consisting of three rectangular steel tubes (internal cross-section: 20 mm × 10 mm; height: 90 mm; wall thickness: 1 mm) welded side by side. The cross-sectional dimensions defined the lateral sampling positions from the root, while the extended height ensured complete vertical penetration of the sample. The sampling depth for each soil sample was 80 mm, and all sampling was performed on the side opposite the sliding sidewall of the sample box. The three soil columns extracted by the sampler were separately pushed into three aluminum boxes and oven-dried at 105 °C for 24 h. This allowed for quantification of water content variations at three parallel positions in the soil samples, from the near-root zone (a) to the far-root zone (c), corresponding to distances of 1–11 mm, 13–23 mm, and 25–35 mm from the root.

#### 2.4.5. NMR Test

The NMR test was conducted using a medium-sized low-field NMR analyser (MesoMR12-110H-I, Suzhou Niumag Analytical Instrument Corporation, Suzhou, China). The system consists of a temperature control unit, an NMR measurement unit, and a data acquisition and analysis system. The magnetic field strength of the instrument was 0.3 ± 0.03 Tesla, with the magnet temperature maintained at 32 ± 0.01 °C, and the probe coil diameter was 25 mm. The prepared cylindrical soil samples were placed into the NMR instrument, and the Carr-Purcell–Meiboom–Gill (CPMG) pulse sequence was used to measure the NMR signal intensity of each sample at 5 cooling temperature points (20 °C, 15 °C, 10 °C, 5 °C, 0 °C) and 11 thawing temperature points (−15 °C, −10 °C, −7.5 °C, −5 °C, −3 °C, −2 °C, −1.5 °C, −1 °C, −0.5 °C, 0 °C, and 5 °C). At each set temperature point, the sample temperature was maintained for at least 30 min [55] before acquiring the NMR signal. The raw NMR signal data collected were processed using the inversion module in the data analysis software [56] to obtain the transverse relaxation time (T_2_) distribution curves of each sample at the corresponding temperatures.

In this study, the paramagnetic regression line (PRL) method was used to calculate the unfrozen water content in the soil, as it has been recommended by previous researchers as a cost-effective and sufficiently accurate approach [57,58]. The specific procedure is as follows: first, based on the NMR initial signal (i.e., the first echo signal) obtained at a series of positive temperatures (20 °C, 15 °C, 10 °C, 5 °C, and 0 °C), a linear relationship between signal intensity and temperature was established, referred to as the paramagnetic regression line. The relationship is expressed as follows:

(2) $$M\left(T\right)=aT+b$$

where $M\left(T\right)$ is the NMR initial signal intensity at temperature T, and a and b are the linear regression coefficients.

Subsequently, the NMR initial signal intensity $M_{0}\left(T_{1}\right)$ of soil samples was measured at various thawing temperature points (from −15 °C to 5 °C). Assuming that Equation (2) remains valid in the sub-zero temperature range, the unfrozen water content $\omega_{u}$ at temperature $T_{1}$ can be calculated by:

(3) $$\omega_{u}=\frac{M_{0}\left(T_{1}\right)}{M\left(T_{1}\right)}\omega_{0}=\frac{M_{0}\left(T_{1}\right)}{aT_{1}+b}\omega_{0}$$

where $\omega_{0}$ is the initial water content (%) of the soil sample, and $\omega_{u}$ is the unfrozen water content (%) at temperature $T_{1}$.

Notably, both the NMR initial signal intensity and the T_2_ peak area exhibit a linear positive correlation with soil water content. However, compared to the T_2_ peak area, the initial signal intensity is less susceptible to temperature-induced interference. Therefore, in this study, the initial signal intensity was selected as the quantitative indicator for calculating unfrozen water content. In addition, the PRL method requires at least four positive temperature measurement points to ensure fitting accuracy [58]; therefore, five positive temperature points were used in this study to enhance the reliability of the regression line fitting.

To evaluate the pore characteristics of soil during the thawing process, this study further analysed the T_2_ distribution curves obtained from NMR measurements during the thawing process of soils with varying water contents. These curves reflect the distribution patterns of pore water at different scales. According to Zhao et al. (2024b) [47], the T_2_ relaxation time can be converted into pore radius r under the assumption of fast diffusion. The conversion relationship is given as follows:

(4) $$r=C_{r}\cdot T_{2}$$

where $C_{r}$ is the pore structure coefficient (μm/ms), which depends on the soil type and is constant for a given soil.

Since this study did not measure the $C_{r}$ value of the samples using mercury intrusion porosimetry (MIP) or other methods, a quantitative analysis of the pore size could not be performed. However, as the soil used in this study is of the same type, $C_{r}$ can be regarded as a constant. Therefore, the variation trend of T_2_ can be used to characterise the relative changes in pore structure. The evolution of T_2_ distribution curves during the thawing process for soils with different water contents can reflect the changes in internal pore size; higher T_2_ values are associated with larger pores, whereas lower values indicate smaller pores.

## 3. Results

### 3.1. Effects of Root Diameter, Temperature and Soil Water Content on Root Pullout Behaviour

#### 3.1.1. Effects of Root Diameter and Temperature on Root Pullout Behaviour

**Figure 6:**
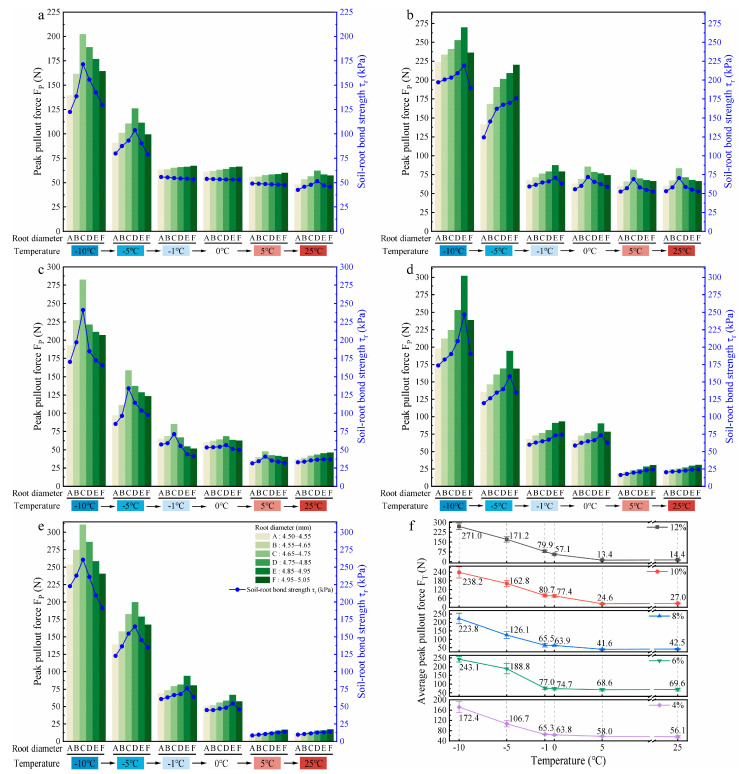
Effects of thawing temperature on root pullout behaviour under different soil water conditions ((**a**–**e**) show the effects of thawing temperature on the peak pullout force (F_P_) and soil–root bond strength ($\tau_{r}$) across six root diameter classes in soils with water contents of 4%, 6%, 8%, 10%, and 12%, respectively; (**f**) shows the effects of thawing temperature on the average peak pullout force (F_T_) across six root diameter classes under five different soil water conditions).

Figure 6a–e illustrate the influence of the thawing process on the peak pullout force (F_P_) and soil–root bond strength ($\tau_{r}$) of roots with six diameter classes in soils with water contents of 4%, 6%, 8%, 10%, and 12%, respectively. At all the values of water content, the F_P_ and $\tau_{r}$ of the six diameter classes of roots showed a similar trend with increasing temperature, both reaching the maximum at −10 °C. The study results determined that $\tau_{r}$ is weakly correlated with root diameter and is primarily influenced by F_P_. Therefore, the following text considers F_P_ as an example to describe the root pullout behaviour during the thawing process.

Across soils with five different water contents, the F_P_ of roots at each temperature point from −10 °C to 0 °C basically increased first and then decreased with root diameter; however, the significance of this trend lessened as temperature increased. Except for the F_P_ of roots in soils with water contents of 6% and 8%, which still maintained this trend even at 5 °C, the F_P_ of roots in the other three soils with water contents increased with root diameter. Only in the soils with low water contents (4% and 6%) did the F_P_ of roots initially increase and then decrease with root diameter with an increase in temperature to 25 °C, while in the soils with high water contents (≥8%), the F_P_ of roots increased with root diameter. Across the soils with varying water content, the F_P_ of roots showed a clear decreasing trend as the temperature rose from −10 °C to 25 °C, and higher soil water contents showed a more pronounced decline. In the low water content (4% and 6%) and high water content (≥8%) soils, the F_P_ of roots tended to stabilize after the temperature rose to −1 °C and 5 °C, respectively. These findings suggest that the relationship between root diameter and F_P_ varies under different combinations of temperature and soil water content.

To clarify the effect of temperature on root pullout behaviour during the thawing process, the parameter of average peak pullout force at a fixed temperature (F_T_) was introduced to analyse the variation pattern of the F_P_ of roots with temperature during thawing, as shown in Figure 6f. Here, F_T_ was obtained by averaging the F_P_ values of six root diameter classes measured at a fixed temperature under each soil water condition. In the soil with 4% water content, the F_T_ of roots gradually decreased as the temperature increased from −10 °C to 25 °C, and reached a minimum of 56.1 N when thawing to 25 °C, which was 67.5% lower than that at −10 °C. In soils with 6%, 8%, 10%, and 12% water contents, the F_T_ of roots showed a trend of first gradually decreasing and then slightly increasing with temperature with 5 °C as the boundary. When thawing to 5 °C, the F_T_ of roots reached the minimum of 68.6 N, 41.6 N, 24.6 N, and 13.4 N, respectively, representing reductions of 71.8%, 81.4%, 89.7%, and 95.1% compared with those at −10 °C. Among them, in soils with 4%, 6%, and 8% water contents, the F_T_ of roots decreased most significantly when thawing from −5 °C to −1 °C, by 38.8%, 59.2%, and 48.1%, respectively. In contrast, in soils with 10% and 12% water contents, the greatest reduction in the F_T_ of roots occurred during the phase transition from ice–water coexistence to fully liquid water (from 0 °C to 5 °C), by 68.2% and 76.5%, respectively; at the same time, the reductions in the F_T_ of roots in soils with 4%, 6%, and 8% water contents were 9.1%, 8.2%, and 34.9%, respectively. These results indicate that the reduction in the F_T_ of roots caused by temperature-induced thawing is evident, and the degree of decrease in the F_T_ of roots during the whole thawing process increases with soil water content. Moreover, the temperature stage where the F_T_ of roots drops sharply also shifts to higher values with increasing soil water content.

#### 3.1.2. Effects of Soil Water Content on Root Pullout Behaviour

**Figure 7:**
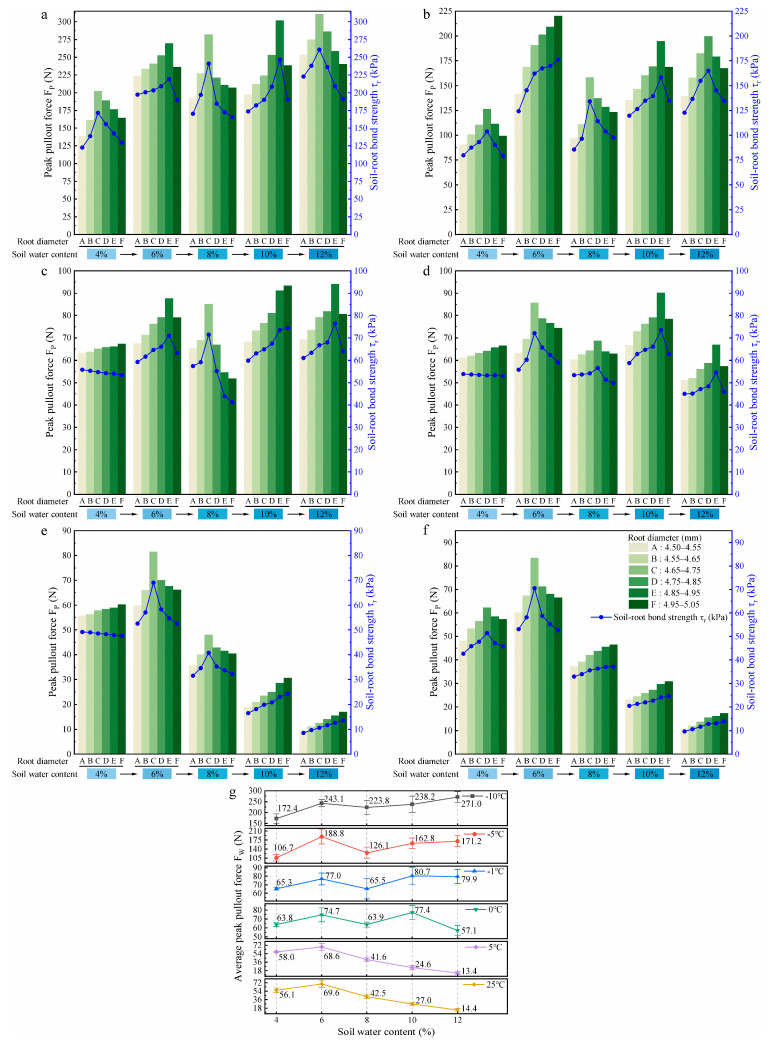
Effects of soil water content on root pullout behaviour under different thawing temperatures ((**a**–**f**) show the effects of soil water content on the peak pullout force (F_P_) and soil–root bond strength ($\tau_{r}$) across six root diameter classes at thawing temperatures of −10 °C, −5 °C, −1 °C, 0 °C, 5 °C, and 25 °C, respectively; (**g**) shows the effects of soil water content on the average peak pullout force (F_W_) across six root diameter classes under six thawing temperatures).

During the thawing process, an increase in soil water content was observed with rising temperature. To examine its influence, root pullout behaviour was evaluated under five water content levels at different temperatures. Figure 7a–f show the effects of five soil water contents on the F_P_ and $\tau_{r}$ of roots with six diameter classes at −10 °C, −5 °C, −1 °C, 0 °C, 5 °C, and 25 °C, respectively.

To elucidate the effect of soil water content on root pullout behaviour during the thawing process, the parameter of average peak pullout force at a fixed soil water content (F_W_) was introduced to analyse the variation pattern of the F_P_ of roots, as shown in Figure 7g. Here, F_W_ was obtained by calculating the average of the F_P_ values of six root diameter classes measured from soils with fixed water content under each thawing temperature.

In the low-temperature slow thawing stage (−10 °C and −5 °C), the F_W_ of roots showed an initial increase, then decreased, and subsequently returned to an increasing trend with the increase in soil water content (from 4% to 12%). The decreasing segments occurred during the increase in soil water content from 6% to 8%, with reductions of 7.9% and 33.2%, respectively. In the soil with 4% water content, the F_W_ of roots reached the minimum values of 172.4 N and 106.7 N, respectively, which were 36.4% and 43.5% lower than the maximum values (soils with 12% and 6% water contents) at −10 °C and −5 °C. In the ice–water intense phase transition stage (−1 °C and 0 °C), with the increase in soil water content, the F_W_ of roots fluctuated slightly, and the maximum reductions occurred during the increase in soil water content from 6% to 8% and from 10% to 12%, with values of 14.9% and 26.2%, respectively. At 10% soil water content, the F_W_ of roots reached maximum values of 80.7 N and 77.4 N, while minimum values of 65.3 N and 57.1 N were observed in soils with 4% and 12% water contents. Compared with the maximum values (soil water content of 10%), the differences were 19.1% and 26.2%, respectively. In the liquid water stage (5 °C and 25 °C), with 6% soil water content as the boundary, the F_W_ of roots increased slightly first and then decreased steadily with the rising soil water content from 4% to 12%. Notably, significant reductions of F_W_ were observed between 10 and 12% soil water content, 45.5% and 46.7%, respectively. The F_W_ of roots reached the minimum at the soil water content of 12%, which were 13.4 N and 14.4 N, respectively, 80.5% and 79.3% lower than those at the soil water content of 6%. These results indicate that the increase in soil water content significantly affects the F_W_ of roots and the soil water content interval that causes a sharp decline in the F_W_ of roots shifts toward higher water content levels as the temperature increases. In the low-temperature range (−10 °C and −5 °C), the anchorage of roots at less water content (4%) in the soil is much weaker than in high water content (12%) soil, but this pattern gradually reverses as the temperature rises to the positive temperature range.

#### 3.1.3. Evaluation of the Main Influencing Factors on Root Pullout Behaviour

To accurately evaluate the degree of influence of root diameter, temperature, and soil water content on the F_P_ of roots, the previously stated results were considered. We also considered the significant water phase change phenomenon (ice to water) that occurs during the temperature rise process, which will amplify the influence of temperature on the pullout force of roots. These aspects interfere with the effects of the other two factors. This study divided the entire thawing process into two typical temperature stages: the ice–water coexistence stage (−10 °C to 0 °C) and the liquid water stage (5 °C to 25 °C), and conducted multiple linear regression analyses separately. This measure allows for clearer identification of the dominant effect of each factor on the mechanical response of roots in different temperature stages. The corresponding multiple linear regression analysis results are shown in Table 5.

In the ice–water coexistence stage (−10 °C to 0 °C), root diameter, temperature, and soil water content have shown significant effects on the F_P_ of roots (*p* < 0.05). Notably, the significance of temperature and soil water content on the F_P_ of roots (*p* < 0.001) was relatively higher than that of root diameter (*p* < 0.05). Concurrently, the standardised regression coefficient (Beta) of temperature was −0.918, and its absolute value was much higher than that of soil water content (Beta = 0.156) and root diameter (Beta = 0.078), indicating that in this temperature stage, temperature change played a dominant role, followed by soil water content. The corresponding regression model is as follows:

(5) $$F_{P}=-129.31+33.829\cdot D-16.756\cdot T+397.045\cdot W\;\;\;\;\;\;\;\;\;\;\;\;\;\;\;\;R^{2}=0.873$$

where F_P_ is the peak pullout force of roots (N), D is the root diameter (mm), T is the temperature (°C), and W is the soil water content (%).

In the liquid water stage (5 °C to 25 °C), the impact of temperature on the F_P_ of roots is significantly weakened (Beta = 0.017, *p* = 0.768), almost negligible; under these conditions, the dominant factor is soil water content (Beta = −0.888, *p* < 0.001), indicating that in a soil environment without ice, soil water content becomes the key variable affecting the mechanical behaviour at the root–soil interface, followed by root diameter (Beta = 0.113, *p* = 0.057). The regression equation for this stage is as follows:

(6) $$F_{P}=27.499+13.682\cdot D+0.035\cdot T-644.112\cdot W\;\;\;\;\;\;\;\;\;\;\;\;\;\;\;\;\;\;R^{2}=0.812$$

The above results demonstrate that temperature variation significantly affects the F_P_ of roots during the ice–water phase transition stage. In contrast, upon entering the liquid water stage, the controlling effect of soil water content emerges as a more prominent factor, while the effect of root diameter is relatively weak in both stages.

### 3.2. Effects of Root–Soil Interface, Soil Water Distribution, Ice–Water Phase States and Pore Structure on Root Pullout Behaviour

#### 3.2.1. Effects of the Root–Soil Interface on Root Pullout Behaviour

**Figure 8:**
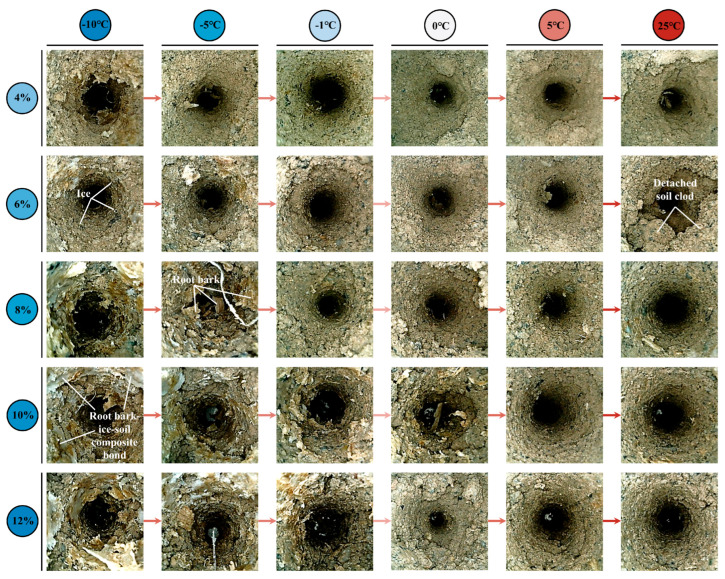
Root–soil interface failure characteristics observed by endoscopy.

Endoscopic observation results (Figure 8) revealed that with the increase in root diameter in low soil water content (4% and 6%) soils at the liquid water stage (5 °C and 25 °C), the root–soil interface gradually transitioned from whole-root sliding to partial soil detachment, forming a composite failure mode. Furthermore, traditional views suggest that root failure in soil usually manifests in two modes: root pullout or root breakage. However, a new root failure mode was discovered through root pullout tests during the ice–water phase transition stage (−10 °C to 0 °C): root bark peeling. Endoscopic observations have shown (Figure 8) that a large amount of ruptured and detached root bark remained in the soil after root pullout during the ice–water phase transition stage. Especially in soils with high water content (≥8%) at −10 °C and −5 °C, these detached root barks formed a distinct root bark–ice–soil composite bonding body with ice crystals and soil particles, significantly altering the frictional characteristics of the root–soil interface.

**Figure 9:**
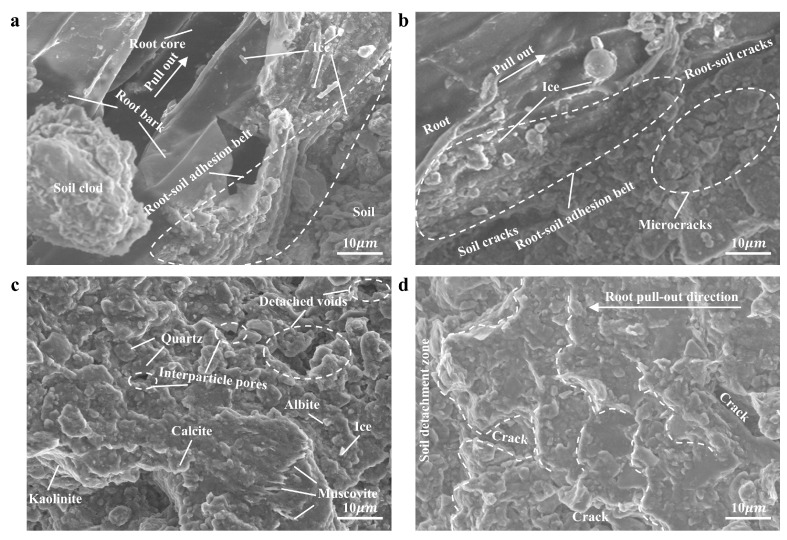
ESEM test results ((**a**) shows the contact relationship between the pulled root and soil; (**b**) shows the contact relationship between the unpulled root and soil; (**c**) shows the undisturbed soil surface; (**d**) shows the disturbed soil surface after root pullout).

To further reveal the microstructural features of this new failure mode, ESEM observation and analysis were conducted on typical samples under −10 °C conditions (Figure 9). Figure 9a presents the root bark and soil particles that formed a tightly bonded root–soil adhesion belt. During pullout, the root bark experienced tensile rupture, and the root core was pulled out from the root bark, leaving the detached root bark in the soil. A distinct gap was observed between the smooth root core and the ruptured root bark indicating the root–soil failure surface shifted to the interface between the ruptured root bark and the root core. Since the combined tensile strength of the root bark and the bonding strength between the root bark and core was much smaller than the root–soil adhesion, the F_P_ of roots was significantly reduced. Figure 9b further shows that ice crystals and soil particles in the root–soil adhesion belt remain tightly bound to the surface of the root bark. During the pullout process, the local soil moved, causing internal soil cracking and simultaneously compressing the upper soil in the direction of root pullout. This further led to multiple microcracks and disturbed pores in the upper soil resulting in separation from the root and development of root–soil cracks. The frictional effect at the root–soil interface was modified from simple root sliding to internal shear failure within the soil, resulting in an overall reduction in frictional resistance at the root–soil interface. A comparison of the microstructural features in Figure 9c,d shows that undisturbed soil particles were disorderly in their arrangement and had rough structure (Figure 9c), with a large potential frictional contact area; whereas the soil surface disturbed by root pullout becomes significantly flattened, and the soil particles are arranged in a layered structure along the root pull-out direction (Figure 9d). In some regions, large-scale detachment is observed, further verifying that the failure mode of the root–soil interface has transformed into internal failure of the soil surrounding the root.

#### 3.2.2. Effects of Soil Water Distribution Characteristics on Root Pullout Behaviour

**Figure 10:**
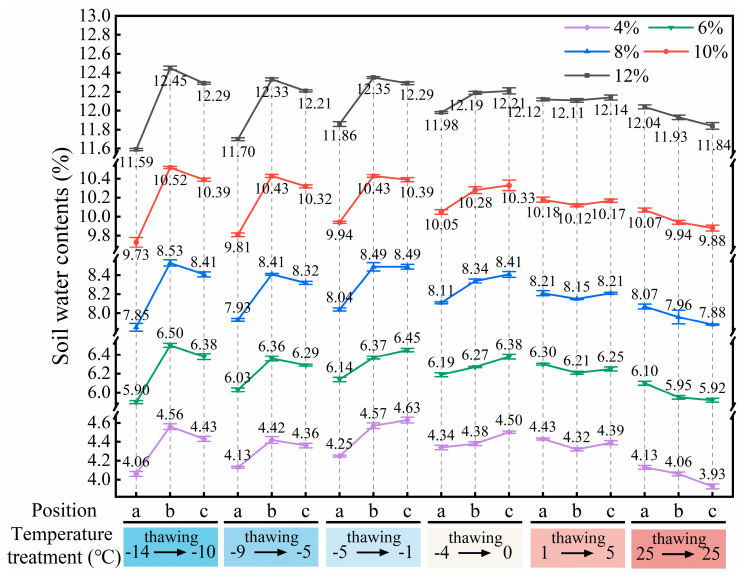
Soil water distribution characteristics after root pullout under different temperature treatments (a, b, and c indicate soil sampling positions at distances of 1–11 mm, 13–23 mm, and 25–35 mm from the root, respectively).

As shown in Figure 10, under the designed thawing test conditions for the ice–water phase transition (−10 °C to 0 °C), soil water migration was mainly controlled by low-temperature (−14 °C to −4 °C) freezing. The soil at the far-root zone (position c) was closest to the low-temperature circulating fluid and underwent preferential freezing; driven by the water potential gradient and the extrusion effect generated by ice crystal growth, soil water migrated from the near-root zone (position a) to the far-root zone, resulting in the lowest soil water content at position a (Figure 10), which enhanced the matric suction at the root–soil interface, manifesting as higher F_T_ of roots (Figure 6f). As the freezing temperature increased, the soil water content at position a gradually increased, leading to a weakening of matric suction and a corresponding decrease in F_T_ of roots (Figure 6f). In addition, different freezing temperatures altered the aggregation location of soil water, thereby regulating the failure characteristics of the root–soil interface. When the root–soil composite was under lower freezing temperatures (−14 °C and −9 °C), a distinct freezing front was formed in the middle zone (position b), where water and ice crystals concentrated (Figure 10), significantly increasing the soil strength at that zone, making the soil less prone to detachment failure during root pullout (Figure 8). As the freezing temperature of the root–soil composite increased (−5 °C and −4 °C), the freezing front gradually migrated toward the far-root zone, resulting in the high water content zone shifting to position c (Figure 10), and the difference in soil water (ice) content between the near-root zone and the middle zone (position a to b) gradually decreased, leading to a reduction in soil strength around the roots and making partial soil detachment more likely during root pullout (Figure 8), thus decreasing the F_T_ of roots (Figure 6f). Under the liquid water test conditions (5 °C to 25 °C), soil water migration was jointly influenced by environmental temperature, root water absorption, air vapour condensation, or soil water evaporation. Under the test condition of lower liquid water temperature (5 °C), the condensation effect of air vapour dominated, and root water uptake and the water migration effect driven by environmental temperature (1 °C) tended to reach a balance. The soil water distribution in each water content condition was relatively uniform and overall higher than the initial set value (Figure 10). At the same time, this phenomenon also explains that in the soil with 4% initial water content, the F_T_ of roots under the 5 °C test condition was higher than that under the 25 °C test condition, while in soils with initial water contents ≥ 6%, the F_T_ of roots under the 5 °C test condition was the lowest throughout the entire thawing process (−10 °C to 25 °C) (Figure 6f). When the temperature increased to the 25 °C test condition, root water uptake and soil water evaporation became the dominant factors, showing that the soil water content around the roots (position a) was higher than that at positions farther from the roots (b and c) (Figure 10). This water distribution pattern (a > b > c) promoted the increase in soil brittleness in areas away from the roots (positions b and c), making partial soil detachment more likely to occur (Figure 8), and thus reducing the F_T_ of roots (Figure 6f).

#### 3.2.3. Effects of Ice–Water Phase States and Pore Structure on the Pullout Behaviour of Roots

**Figure 11:**
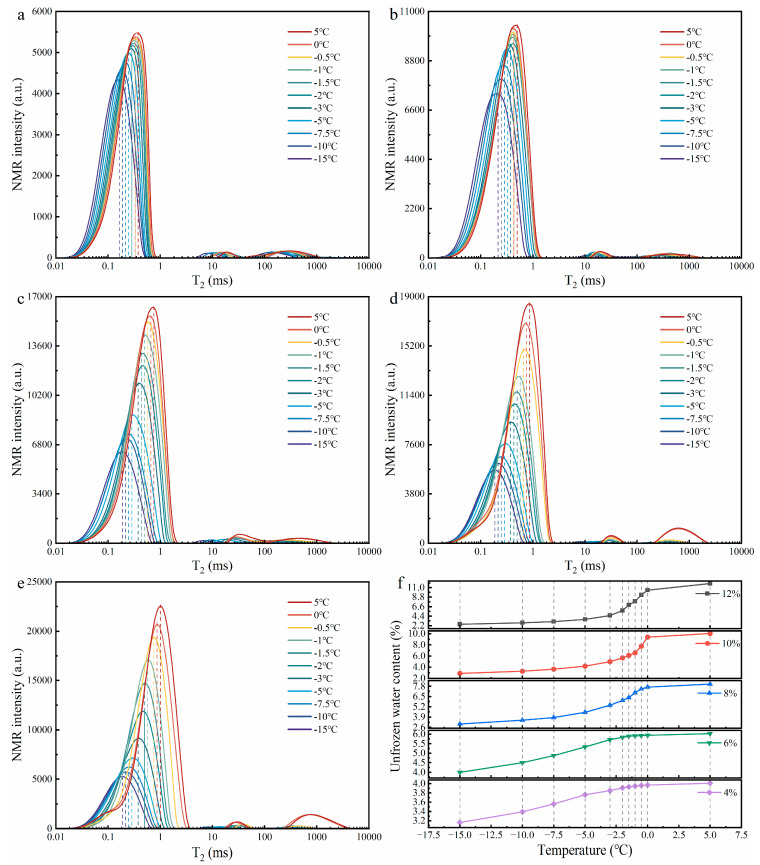
NMR test result diagrams ((**a**–**e**) show the T_2_ distribution curves at 11 thawing temperature points for soil samples with initial water contents of 4%, 6%, 8%, 10%, and 12%, respectively; (**f**) shows the variation in unfrozen water content during the thawing process in soil samples with different initial water contents).

Figure 11a–e show the T_2_ distribution curves at 11 thawing temperature points for soil samples with initial water contents of 4%, 6%, 8%, 10%, and 12%, respectively. The unfrozen water content calculated using Equation (3) (Figure 11f) indicates that as temperature increased, the unfrozen water content in soils of all initial water contents gradually increased, resembling a wetting process—namely, the increase in free water in the soil during thawing reduced the matric suction at the root–soil interface, which was manifested as a decrease in the F_T_ of roots (Figure 6f).

The ice content in soil inferred from the unfrozen water content (Table 6) further reveals that the ice content in soils with various initial water contents decreased significantly with increasing temperature, and the evolution trend of ice content differed among soils with different initial water contents. In soils with initial water contents of 4%, 6%, and 8%, the largest decreases in ice content occurred during thawing from −5 °C to −1 °C, with reductions of 76.0%, 82.9%, and 69.6%, respectively, while in soils with initial water contents of 10% and 12%, the decrease in ice content continued to grow with increasing temperature. Compared with the variation trend of the F_T_ of roots with temperature in Figure 6f, it can be seen that the variation trend of the F_T_ of roots is highly consistent with the variation trend of ice content: in soils with initial water contents of 4%, 6%, and 8%, the F_T_ of roots decreased the most during thawing from −5 °C to −1 °C, while in soils with initial water contents of 10% and 12%, the F_T_ of roots significantly decreased during thawing from −10 °C to 5 °C, and both decreased the most during thawing from 0 °C to 5 °C. This phenomenon indicates that soil ice content dominated the mechanical properties of the root–soil interface during the ice–water phase transition stage.

In addition, with the variation in T_2_ spectra in Figure 11a–e, it can be observed that with increasing temperature, the T_2_ curve amplitude of the five soil samples with different initial water contents gradually increased and shifted significantly to the right, indicating that the compressive force released by ice crystal melting led to an increase in both the number and size of soil pores and the structure tended to loosen. Pore enlargement led to a decrease in both root–soil and soil–soil bonding strength. This soil structure loosening process not only reduced the F_T_ of roots (Figure 6f) but also increased the occurrence of partial soil detachment during root pullout (Figure 8).

## 4. Discussion

### 4.1. The Unique Root Pullout Behaviour During Thawing

Previous studies on root pullout force were all conducted at temperatures above 0 °C (i.e., under non-freezing conditions), and it is generally believed that the F_P_ of roots increases monotonically with root diameter, owing to the assumption that larger root diameters can provide greater root–soil contact area [23,59]. However, the results of this study at the liquid water stage (5 °C and 25 °C) (Figure 7e,f) demonstrated that only under specific soil water content conditions (e.g., 4%, 10%, and 12% at 5 °C; high soil water content ≥ 8% at 25 °C), the F_P_ of roots increased monotonically with root diameter. Under other conditions of the liquid water stage and ice–water phase transition stage (−10 °C to 0 °C) (Figure 7a–d), the F_P_ of roots followed the trend of initial increase followed by a decrease with increasing root diameter. This non-monotonic change should be closely related to the shift in the failure mode at the root–soil interface. This transition is attributed to the fact that when the root diameter exceeds the rupture threshold diameter of the soil, the frictional force at the root–soil interface exceeds the shear strength of the local soil, causing the failure surface to shift into the soil [28,60]. The resistance to slippage at the soil–soil interface is lower than that at the root–soil interface [61,62], thereby reducing the overall pullout resistance at the root–soil interface. Particularly, under dry (low soil water content) soil conditions, the brittle characteristics of soil become more pronounced [63,64], making this failure mode transition more significant, resulting in a non-monotonic increasing trend of the F_P_ of roots with increasing root diameter.

As supplementary evidence, macroscopic endoscopic observations (Figure 8) and the microscopic ESEM observations (Figure 9) reveal that during the ice-water phase transition stage, the root–soil interface failure mode with increasing root diameter was characterised by the combined action of root bark peeling and partial soil detachment around the root, and exhibited different transitions with temperature and soil water content. In soils with high water content (≥8%) during the slow phase transition stage (−10 °C and −5 °C), the root bark peeling failure mode was prominent, due to the enhanced soil bonding strength under high ice content [65]. In contrast, in soils with relatively low water content (<8%) during the intense phase transition stage (−1 °C and 0 °C), the failure mode was more dominated by partial soil detachment around the root, reflecting the prominent soil brittleness. The above two failure modes highlight the non-monotonic relationship between root diameter and the F_P_ of roots.

### 4.2. The Significance of Factors Influencing Root Pullout Behaviour During Thawing

Previous studies have confirmed that soil water content and root diameter are important factors influencing root pullout resistance [22,66]. However, the study (Section 3.1.3) has revealed an interesting statistical pattern: during the ice–water coexistence stage (−10 °C to 0 °C), temperature plays a dominant role in regulating root pullout behaviour; in the liquid water stage (5 °C to 25 °C), the influence of temperature became almost negligible, while soil water content exhibited a significantly enhanced effect on the F_P_ of roots; in both stages, root diameter has the weakest effect. These findings highlight a shift in the controlling mechanisms across thawing stages. During the ice–water coexistence stage, as temperature increased from −10 °C to 0 °C, the F_T_ of roots in soils with different water contents sharply decreased by more than 63% (Figure 6f). This decline was primarily driven by the melting of cementing ice at the root–soil interface, accompanied by the reduction in matric suction and pore enlargement. NMR tests provided critical supporting evidence: the trend of F_T_ reduction closely matched the decline in ice content observed in Table 6. This finding is consistent with Wang et al. (2024b) [67] and Yang et al. (2024b) [68], who reported a linear positive correlation between ice content and ice-bonding cohesion. As temperature rose, unfrozen water content progressively increased (Figure 11f), resembling a wetting process that weakened matric suction at the root–soil interface [69]. Furthermore, the failure characteristics of the root–soil interface observed by endoscopy (Figure 8) and environmental scanning electron microscopy (Figure 9) confirmed that the cemented structure formed by ice crystals significantly enhanced the interfacial bonding strength [70]. In addition, T_2_ spectral analysis (Figure 11a–e) indicated that the release of compressive force from melting ice crystals resulted in pore enlargement and structural loosening of the soil [71,72], which further reduced the bonding strength at the root–soil interface [73]. These pieces of evidence collectively explain that, during the ice–water coexistence stage, the temperature-induced reduction in ice content was the dominant mechanism controlling the mechanical response at the root–soil interface. In the liquid water stage (5 °C to 25 °C), the F_W_ of roots exhibited a non-monotonic trend with increasing soil water content, initially increasing and then decreasing (Figure 7g), consistent with recent findings by Zhang et al. (2020) [74] and Zhu et al. (2022b) [54]. This phenomenon can be explained as follows: under dry conditions (4% water content), the soil exhibited low cohesion and high brittleness [63,75], making the soil surrounding the root more prone to partial detachment failure (Figure 8). As soil water content increased to 6%, stable water films formed between soil particles enhanced interfacial bonding strength and friction at the root–soil interface [75]. However, when soil water content exceeded a critical threshold (6%), increased pore water pressure and reduced matric suction weakened the cohesion of the root–soil interface [76], while the excess water acted as a lubricant, further reducing bonding strength [49]. Additionally, the results of the soil water migration test (Figure 10) showed that water redistribution around the root zone became increasingly important under liquid-phase conditions [54,66]. Influenced by both environmental temperature [77] and root water uptake [78], more water accumulated in the near-root zone (position a). In soils with 4% water content, this redistribution enhanced interfacial cohesion. Conversely, in soils with ≥6% water content, it led to excessive wetting near the root, weakening the interfacial bonding strength. Additionally, this redistribution also caused higher moisture levels in the rhizosphere compared to zones farther from the root, increasing the brittleness of distant soil, aggravating partial soil detachment (Figure 8), thereby reducing root pullout resistance. Moreover, root diameter exhibited the weakest influence on root pullout behaviour across both temperature ranges. Although previous studies have often linked larger root diameters to enhanced pullout resistance due to increased root–soil contact area [22,79], this relationship was greatly diminished under thawing conditions. This may be attributed to changes in root–soil failure modes and soil shear properties at low temperatures, as confirmed by the ESEM analysis (Figure 9).

According to relevant studies, it is easy to understand that an increase in temperature can significantly weaken root reinforcement, and this weakening becomes more pronounced with increasing soil water content [80,81]. However, as temperature rises, our findings revealed that the critical temperature at which the F_P_ of roots decreases varies across different soil moisture levels. In soils with low water content (4% and 6%) and high water content (≥8%), the F_P_ of roots tends to stabilize when the temperature reaches −1 °C and 5 °C, respectively (Figure 6a–e). This difference may be related to soil ice content and the sequential melting behaviour of pore ice. NMR results provide strong supporting evidence for this: Table 6 shows that both ice content and the degree of freezing increase with rising soil water content during the thawing process, which is consistent with the findings of Wei et al. (2025) [82] and Ren et al. (2025a) [83]. In addition, the T_2_ distribution curves in Figure 11a–e exhibit a clear rightward shift as soil water content increases. At the 11 thawing temperature points, the average T_2_ value corresponding to the first signal peak increases from 0.275 ms at 4% water content to 0.501 ms at 12%, with lower T_2_ values representing smaller pore sizes and higher values indicating larger pores [47]. This suggests that higher water content leads to the formation of significantly larger pore ice. Considering these NMR findings with endoscopic observations (Figure 8), it can be inferred that in low water content soils (4% and 6%), the relatively low ice content and weak development of ice bonds (Figure 8), along with early melting of smaller pore ice [73], contribute to the earlier stabilization of the F_P_ of roots. In contrast, in high water content soils (≥8%), the higher ice content and greater degree of freezing promote the formation of extensive ice bridges (Figure 8), which require more energy to melt [84]. Moreover, larger pore ice melts more slowly [73], thereby delaying the arrival of the stabilization point. Within the corresponding range of water content and temperature, the F_P_ of roots demonstrates a stable trend and serves as a valuable reference for numerical simulations and slope protection design utilizing vegetation in cold regions [85,86]. For example, when evaluating the stability of *A. sparsifolia* root–clayey sand composite slopes, considering the temperature and humidity thresholds at the two stable points of −1 °C and 5 °C will result in greater accuracy.

A novel failure mode—root bark peeling—was identified through root pullout tests, macroscopic endoscopic observations (Figure 8), and microscopic ESEM observations (Figure 9). This indicates that in low-temperature frozen environments, root bark strength and its adhesion to the root core play a vital role in the F_P_ of roots. Specifically, under low temperatures (particularly high moisture content conditions), the volumetric expansion of ice increases the bonding between ice–soil layers and roots [87,88], thereby enhancing the root–soil interfacial forces. When the applied tensile stress exceeds a critical threshold, root bark detachment occurs. These findings suggest that in cold and arid regions, particularly in snow-covered high-slope areas, selecting vegetation with strong root bark integrity and strong adhesion between the root bark and root core is essential for preventing slope failures caused by the self-weight of accumulated snow [89,90].

Additionally, the thawing process, characterised by ice–water phase transitions, sharply increases the soil liquid water content [91,92]. NMR results (Figure 11f) indicate that as the temperature increases from −10 °C to 5 °C, when the initial soil water content increases from 4% to 12%, the increase rate of liquid water content rises from approximately 18.4% to over 316.7%, leading to an increase in the reduction rate of the F_T_ of roots from about 66.4% to 95.1% (Figure 6f). In cold and arid regions, although soil water is essential for the growth of desert vegetation [93,94], rapid and large volumes of water influx during snowmelt or rainfall periods pose significant threats to slope stability [95,96]. Therefore, given the high frequency and severity of snowmelt- or rainfall-induced landslides in cold and arid regions [97,98,99], controlling slope moisture content during these critical periods is crucial. It is recommended that effective water management strategies be incorporated into slope stabilization and ecological restoration practices to mitigate the risk of slope instability in such environments.

The present study simulated a single freeze–thaw process under controlled laboratory conditions, which, although valuable for isolating key variables, does not capture the complexity of actual field environments. In our future work, we plan to conduct field-scale root pullout experiments using in situ soil monitoring systems under coupled conditions of snowmelt infiltration, multiple freeze–thaw cycles, and rainfall–thawing scenarios. This will help validate and extend the findings of the current study, allowing for a more realistic assessment of the long-term evolution of root–soil interfacial strength under complex climatic conditions. At the same time, future studies will explore the biochemical composition of roots—such as lignin and cellulose content—which significantly influence root tensile strength and interfacial cohesion, thereby affecting slope stability [100]. Moreover, although Young’s modulus was not evaluated in this study, future research will involve low-temperature single-root tensile tests to accurately quantify this parameter under varying temperature and water conditions [101].

## 5. Conclusions

In this study, targeting the context of vegetation root reinforcement on slopes in freeze–thaw environments in cold and arid regions, remoulded root–soil composites were constructed using *A. sparsifolia* roots and clayey sand. The root pullout behaviour and root–soil interface mechanisms during the thawing process were systematically analysed. The main conclusions are as follows:

(1) The temperature, water content, and diameter are significant factors that influence root pullout behaviour. During the ice–water coexistence stage, which spans from −10 °C to 0 °C, temperature changes are the dominant influence. In the liquid water stage, ranging from 5 °C to 25 °C, the primary factor is soil water content. In both stages, the effect of root diameter is the weakest.
(2) Rising temperature significantly weakens the root reinforcement effect, and the weakening becomes more severe with increasing soil water content. As the temperature increased from −10 °C to 25 °C and soil water content increased from 4% to 12%, these changes led to a maximum reduction in the average peak pullout force (F_T_) of roots exceeding 95%. During the ice–water phase transition stage (−10 °C to 0 °C), the F_T_ of roots sharply decreased by more than 63%. Thus, these findings indicated that the sharp decline in root reinforcement capacity during the thawing period should be taken into account in slope engineering design in cold and arid regions.
(3) Regarding the rapid decline in root reinforcement capacity during thawing, this study reveals that in soils with low water content (4–6%), the peak pullout force (F_P_) of roots tended to stabilize at −1 °C, while in soils with high water content (≥8%), stabilization occurred when the temperature exceeded 5 °C. This indicates that the F_P_ values obtained at ≤−1 °C (low water content), ≤5 °C (high water content) and normal temperature are highly significant for the protection engineering of clayey sand slopes in arid and cold regions. They can facilitate more straightforward slope stability calculations and enhance the accuracy of numerical analyses.
(4) A new failure mode has been discovered: root bark peeling that occurs under low-temperature conditions (≤0 °C) and high soil moisture conditions (≥8%). This finding suggests that in cold arid regions, especially on high-slope snow-covered areas, employing vegetation with strong root bark integrity and strong adhesion between the root bark and root core is essential for preventing slope failures caused by the weight of snow accumulation or other overburden stress increases.

## Figures and Tables

**Table 1:** Basic physical properties of the test roots and soil.

Basic physical parameters of the experimental root	Natural water content	64.50%
	Tissue density	0.780 g/cm^3^
	Maximum water absorption	385.30%
	Maximum longitudinal shrinkage	12.43%
	Maximum radial shrinkage	52.38%
Main physical properties of experimental soil	Natural water content	8.15%
	Natural dry density	1.576 g/cm^3^
	Optimum water content	11.23%
	Maximum dry density	1.97 g/cm^3^
	Cohesion	7.68 kPa
	Friction angle	15.26°
	Liquid limit	27.15%
	Plastic limit	12.03%
	Plasticity index	15.12
	Diameter 10% weigh sift	0.0037 mm
	Diameter 30% weigh sift	0.0103 mm
	Diameter 60% weigh sift	0.1169 mm
	Uniformity coefficient	31.59
	Coefficient of curvature	0.25
	USCS classification	Clayey sand (SC)

**Table 2:** Design scheme for root pullout tests during the thawing process.

Parameter	Value
Pullout speed (mm/min)	20
Root diameter (mm)	4.50–4.55, 4.55–4.65, 4.65–4.75, 4.75–4.85, 4.85–4.95, 4.95–5.05
Root embedment depth (mm)	80
Soil water content (%)	4, 6, 8, 10, 12
Soil dry density (g/cm^3^)	1.576
Root–soil composite volume (cm^3^)	512
Test temperature (°C)	−10, −5, −1, 0, 5, 25
Pre-freezing temperature (°C)	−14, −9, −5, −4, 1, 25
Freezing time (h)	4
Total number of samples	180

**Table 3:** Test schemes for the four mechanism analysis methods.

Test Type	Parameter	Value
Endoscopic imaging test	Root diameter (mm)	4.95–5.05
	Soil water content (%)	4, 6, 8, 10, 12
	Thawing temperature (°C)	−10, −5, −1, 0, 5, 25
	Total number of samples	30
ESEM test	Soil water content (%)	12
	Environmental temperature (°C)	−10
	Total number of samples	3
Soil water migration test	Soil water content (%)	4, 6, 8, 10, 12
	Thawing temperature (°C)	−10, −5, −1, 0, 5, 25
	Sampling position	a, b, c
	Sampling depth (mm)	80
	Total number of samples	90
NMR test	Soil water content (%)	4, 6, 8, 10, 12
	Soil dry density (g/cm^3^)	1.576
	Thawing temperature (°C)	−15, −10, −7.5, −5, −3, −2, −1.5, −1, −0.5, 0, 5
	Total number of samples	5

Notes: In the soil water migration test, a, b, and c refer to sampling positions at distances of 1–11 mm, 13–23 mm, and 25–35 mm from the root, corresponding to the near-root zone, middle zone, and far-root zone, respectively. A total of 90 samples includes three parallel measurements for each condition.

**Table 4:** Corresponding relationship between pre-freezing temperature and test temperature for root–soil composites.

Parameter	Condition 1	Condition 2	Condition 3	Condition 4	Condition 5	Condition 6
Pre-freezing temperature (°C)	−14	−9	−5	−4	1	25
Room-temperature thawing	⬇	⬇	⬇	⬇	⬇	⬇
Test temperature (°C)	−10	−5	−1	0	5	25

Note: The downward arrow (⬇) indicates the process of room-temperature thawing.

**Table 5:** Multiple linear regression analysis results of three influencing factors at different thawing stages.

Thawing Phase	Variable	Unstandardized Coefficient (B)	Std. Error	Standardized Coefficient (Beta)	*p*-Value	R^2^	F Value
Ice–water coexistence (−10 °C to 0 °C)	Constant	−129.310	68.555	-	0.062	0.873	266.932 **
	Root diameter	33.829	14.317	0.078	0.020 *		
	Temperature	−16.756	0.603	−0.918	0.000 **		
	Soil water content	397.045	83.883	0.156	0.000 **		
Liquid water phase (5 °C to 25 °C)	Constant	27.499	33.919	-	0.421	0.812	80.700 **
	Root diameter	13.682	7.044	0.113	0.057		
	Temperature	0.035	0.119	0.017	0.768		
	Soil water content	−644.112	42.067	−0.888	0.000 **		

Notes: * *p* < 0.05, ** *p* < 0.01. R^2^ indicates the explanatory power of the regression model for variations in the F_P_ of roots; F value tests the overall significance of the regression model.

**Table 6:** Ice content during the thawing process in soil samples with different initial water contents.

Soil Sample	Ice Content (%)										
	−15 °C	−10 °C	−7.5 °C	−5 °C	−3 °C	−2 °C	−1.5 °C	−1 °C	−0.5 °C	0 °C	5 °C
12%	9.42	9.09	8.81	8.31	7.45	6.25	5.02	4.11	2.68	1.56	0.00
10%	7.14	6.73	6.38	5.86	5.05	4.38	3.93	3.45	2.28	0.65	0.00
8%	5.02	4.55	4.21	3.55	2.65	2.05	1.69	1.08	0.60	0.38	0.00
6%	2.03	1.52	1.15	0.70	0.32	0.19	0.14	0.12	0.11	0.08	0.00
4%	0.85	0.62	0.45	0.25	0.16	0.10	0.08	0.06	0.05	0.04	0.00

## Data Availability

Data are contained within the article.
